# Stillbirth is associated with perceived alterations in fetal activity – findings from an international case control study

**DOI:** 10.1186/s12884-017-1555-6

**Published:** 2017-11-13

**Authors:** Alexander E. P. Heazell, Jane Warland, Tomasina Stacey, Christin Coomarasamy, Jayne Budd, Edwin A. Mitchell, Louise M. O’Brien

**Affiliations:** 1 St Mary’s Hospital, Manchester University NHS Foundation Trust, Manchester Academic Health Science Centre, M13 9WL, Manchester, UK; 2 Maternal and Fetal Health Research Centre, School of Medical Sciences, Faculty of Biology, Medicine and Health, University of Manchester, Manchester, UK; 3 Mother’s Babies and Families Research Group, School of Nursing and Midwifery, University of South Australia, Adelaide, Australia; 4 School of Healthcare, University of Leeds, Leeds, UK; 5 Department of Paediatrics: Child and Youth Health, University of Auckland, Auckland, New Zealand; 6 Sleep Disorders Center and Department of Obstetrics & Gynecology, University of Michigan, Ann Arbor, MI USA

**Keywords:** Maternal perception, Fetal movement, Reduced fetal movement, Exaggerated fetal movement, Stillbirth

## Abstract

**Background:**

Stillbirth after 28 weeks gestation affects between 1.3–8.8 per 1000 births in high-income countries. The majority of stillbirths in this setting occur in women without established risk factors. Identification of risk factors which could be identified and managed in pregnancy is a priority in stillbirth prevention research. This study aimed to evaluate women’s experiences of fetal movements and how these relate to stillbirth.

**Methods:**

An international internet-based case–control study of women who had a stillbirth ≥28 weeks’ gestation within 30 days prior to completing the survey (*n* = 153) and women with an ongoing pregnancy or a live born child (*n* = 480). The online questionnaire was developed with parent stakeholder organizations using a mixture of categorical and open–ended responses and Likert scales. Univariate and multiple logistic regression was used to determine crude (unadjusted) and adjusted odds ratios (aOR) with 95% confidence intervals (CI). Summative content analysis was used to analyse free text responses.

**Results:**

Women whose pregnancy ended in stillbirth were less likely to check fetal movements (aOR 0.54, 95% CI 0.35–0.83) and were less likely to be told to do so by a health professional (aOR 0.55, 95% CI 0.36–0.86). Pregnancies ending in stillbirth were more frequently associated with significant abnormalities in fetal movements in the preceding two weeks; this included a significant reduction in fetal activity (aOR 14.1, 95% CI 7.27–27.45) or sudden single episode of excessive fetal activity (aOR 4.30, 95% CI 2.25–8.24). Cases described their perception of changes in fetal activity differently to healthy controls e.g. vigorous activity was described as “frantic”, “wild” or “crazy” compared to “powerful” or “strong”.

**Conclusions:**

Alterations in fetal activity are associated with increased risk of stillbirth. Pregnant women should be educated about awareness of fetal activity and reporting abnormal activity to health professionals.

**Electronic supplementary material:**

The online version of this article (10.1186/s12884-017-1555-6) contains supplementary material, which is available to authorized users.

## Background

Fetal activity is a well-accepted marker of fetal wellbeing, such that maternal perception of changes in activity can indicate fetal compromise. The most commonly reported change is a perceived reduction in fetal movement. Indeed, reduced fetal movements have long been associated with adverse pregnancy outcomes such as fetal growth restriction and stillbirth [1, 2]; this association is thought to result from placental dysfunction which is evident in cases of reduced fetal movements [3, 4]. However, little is known about other aspects of perceived fetal activity, such as an increase in movement, and associations with adverse outcomes. Recent data from a case–control study and a large international cohort study have both suggested that any significant change in the usual pattern of fetal movement is risk factor for stillbirth [5, 6]. Consequently, it is important to better understand maternal perception of altered fetal activity and whether these perceptions can be used to identify fetuses at highest risk of antepartum stillbirth. To address this, we conducted a case–control study to explore novel risk factors associated with stillbirth. The objective of this manuscript is to report maternally-perceived fetal movements in women who experienced a recent stillbirth compared to a control group of women.

## Methods

This is part of a study which investigated potentially modifiable risk factors for stillbirth in an international population. It involved an international group of researchers and clinicians, the STARS (Study of Trends and Associated Risks for Stillbirth) Consortium, partnered with the Star Legacy Foundation and other stillbirth and parental support groups to conduct a web-based survey of women who had experienced a stillbirth using a nested case–control design with a larger uncontrolled cohort of late stillbirths. We have previously reported on the methods of the cohort study [6]. In reporting this study, guidelines from Strengthening the Reporting of Observational Studies in Epidemiology (STROBE) group were followed [7].

### Participants

Participants were recruited to this study by web-based advertising, social media, and word of mouth between September 2012 and August 2014. All participants completed the survey, which asked a range of questions including their recollection of fetal activity. Cases were women, at least 18 years of age, who were fluent in reading and writing English, who had delivered a singleton stillborn baby with no evidence of congenital anomaly at or greater than 28 weeks gestation less than 30 days prior to completing the survey. Controls were either still pregnant (greater than 28 weeks) or had recently delivered a living baby less than 30 days before they completed the survey. Controls also had to be 18 years or older and fluent in English. For both cases and controls those with multiple pregnancies, neonatal death, or fetal loss/live birth prior to 28 weeks gestation were excluded.

### Justification of sample size

To detect a difference in odds ratio of ≥3.0 between women with stillbirths compared to healthy controls, 130 cases would be needed if the exposure had a frequency of 50% and 144 if the exposure had a frequency of 20%, assuming a Power of 80%, *p* = 0.05 and 1:1 matching.

### Survey

The survey included open-ended responses, categorical responses, such as yes/no/don’t know, Likert scales, and selection of responses from a list either through drop-down menus that allowed single responses or check boxes that allowed multiple responses. Participants were invited to describe symptoms and experiences in more detail in accompanying free-text boxes. The format of the final survey included branching logic such that participants were directed through different paths based on their response. The questionnaire is available as a Additional file 1.

### Ethical approval

This study was approved by the Institutional Review Board (IRB) of the University of Michigan (HUM#00063655). Prior to gaining access to the survey participants were informed about the purpose of the study (to look for trends and risk-factors for stillbirth) as well as contact information for a stillbirth support group (First Candle) if they became distressed whilst completing the survey. Informed consent was gained by the participant clicking an “I agree” button prior to gaining access to the survey.

### Data analysis

Data were cleaned by two authors (JW and LO) prior to being sent to CC for analysis. Women found not to fit the inclusion criteria above were excluded from analysis. In addition, there were several instances where women had attempted to complete the survey but did not finish and then returned to complete the survey later. In these cases, only the complete version was retained. Categorical variables were reported as counts and proportions whereas continuous variables were presented in terms of mean (standard deviation) or median (Interquartile range). All statistical analysis was performed with SAS (version 9.4, SAS Institute, Cary, NC) using cross tabulations, with χ^2^ tests, and logistic regressions to find crude (unadjusted) and adjusted odds ratios (aOR) with 95% confidence intervals (CI). The level of statistical significance was set at *p* < 0.05. A multivariable logistic regression model was developed to include maternal variables along with the maternal perception of fetal movements reported to be associated with increased risk of stillbirth, based on prior knowledge and previous literature (maternal age [8], BMI [9], parity [10], gestation [11], placental site [9, 10] (as reported by the mother from her ultrasound scans) and birth weight percentile [12, 13]); country of respondent (US vs non US) and ethnicity of respondent (Caucasian vs Non-caucasian) were also added to the model as they were significantly different between cases and controls. Birth weight percentile was calculated using GROW software [14]. The birth weight percentile was not included in the final multivariate model as birth weight data were missing for 47% of controls. Interaction terms were tested in the model between birth weight percentile and the maternal perception of fetal movements. A sensitivity analysis was carried out to compare whether incorporating the birthweight percentile altered the findings.

Most questions included an option for free text. However, these data were not rich, because not all participants opted to give any response to these questions and even if they did, typically they wrote just a few words or sentences. This meant that the data are not suitable for thematic analysis. Therefore, these data were analysed using ‘summative content analysis’ outlined by Hsieh and Shannon. [15] Comment data from cases and controls were initially analysed separately. Firstly, data from each question were searched for reoccurrence of words. To maintain rigor two authors (JW and JB) separately read and reread the data for recurring words. The most commonly recurring word or phrase participants used, when giving more detail about their experiences, were identified. Once these words were identified, JW and JB met to discuss them and agreement was reached. Exemplar quotes for each of the identified words or phrases were agreed upon by JW and JB. Percentages are included in the table to indicate the ratio of the recurring words with respect to the total number of responses for that question.

## Results

In total, 153 cases and 480 controls completed the survey. Median duration of time since the stillbirth was 13 days (range 1–29 days). Demographics of the sample, including the participant’s country of residence, are shown in Table 1. The mean age of women at the time of stillbirth was 31 (standard deviation 5.4) and was not different between groups. The proportion of Caucasian women was lower in the cases compared to controls (79.7% vs. 90.2% *p* < 0.0002). Although both the United States and Canada had similar proportions of cases and controls, control women were over-represented in respondents from the United Kingdom compared to the proportion of cases (see Table 1). The median gestation at the time of the stillbirth was 37 weeks (range 28–41 weeks), and 52% of the babies in the population were male. A greater proportion of controls reported they had a high-risk pregnancy than cases (*p* = 0.025).

**Table 1:** Demographic information and pregnancy characteristics of participants

	Case(n = 153)	Control(n = 480)	p-value
Mean Maternal Age (years) (std) ^a^	31 (5.4)	30 (4.8)	0.52
Missing	*n* = 5	*n* = 20	
Maternal Race/Ethnicity			
Caucasian	79.7% (n = 122)	90.2% ( *n* = 430)	0.0002
Hispanic	3.3% (n = 5)	3.8% ( *n* = 18)	
Other	17.0% ( *n* = 26)	6.0% ( *n* = 29)	
Missing	0	3	
Median Maternal BMI (kg/m ^2^ ) (IQR) ^a^	27.1 (23–32.3)	25.2 (22.5–31)	0.07 ^*^
Missing	( *n* = 0)	(n = 18)	
Median Gestational Age (weeks) (IQR)	37 (34–39)	37 (32–39)	0.20 ^*^
Missing	(n = 0)	(n = 4)	
Country			0.0012
United States	79.7% ( *n* = 122)	68.7% ( *n* = 325)	
Canada	7.2% ( *n* = 11)	7.0% ( *n* = 33)	
United Kingdom	5.2% ( *n* = 8)	18.4% ( *n* = 87)	
Other	7.8% (n = 12)	5.9% ( *n* = 28)	
Missing	(n = 0)	( *n* = 7)	
Highest Level of Education			0.63
Postdoctoral education	0% (n = 0)	1.3% ( *n* = 6)	
Doctorate Degree	2.0% (n = 3)	5.0% ( *n* = 24)	
Masters Degree	20.9% (n = 32)	20.8% ( *n* = 99)	
Bachelors Degree	37.3% ( *n* = 57)	36.9% ( *n* = 176)	
Technical/Trade School	3.9% (n = 6)	3.6% (n = 17)	
Associates Degree	9.8% (n = 15)	8.0% ( *n* = 38)	
High School Diploma	20.9% (n = 32)	18.2% (n = 87)	
Some High School	1.3% (n = 2)	2.5% (n = 12)	
Other	3.9% (n = 6)	3.8% (n = 18)	
Missing	(n = 0)	(n = 3)	
Employment During Pregnancy			0.09
Work outside the home (full/part time)	67.1% ( *n* = 102)	60.4% ( *n* = 288)	
Work at home (full/part time)	5.9% (n = 9)	7.8% ( *n* = 37)	
Stay at Home Mom	15.8% (n = 24)	24.5% ( *n* = 117)	
Other	7.2% (n = 11)	4.0% ( *n* = 19)	
Unemployed	4.0% (n = 6)	3.4% ( *n* = 16)	
Missing	(n = 1)	(n = 3)	
High-risk pregnancy	24.8% (n = 38)	35.0% ( *n* = 168)	0.025
Medical condition diagnosed before pregnancy	3.3% (n = 5)	7.9% (n = 38)	
Medical condition diagnosed during pregnancy	12.4% (n = 19)	8.1% ( *n* = 39)	
Previous pregnancy complication	3.3% (n = 5)	7.1% ( *n* = 34)	
Previous pregnancy loss	5.9% (n = 9)	11.9% (n = 57)	

^*^ Non-parametric test (Wilcoxon two-sample t-test)

^a^ Standard deviation (std), Interquartile range (IQR)

Women who experienced a stillbirth reported that they were less likely to keep track of baby’s movements (aOR 0.54 95% CI 0.35–0.83, *p* = 0.005, Table 2) and reported that they were less likely to have been told by their health provider to do so during pregnancy, compared to healthy controls (aOR 0.55, 95% CI 0.36–0.86, *p* = 0.008). A perception of increased frequency and/or strength of fetal movements in the previous two weeks was protective against stillbirth (aOR 0.36, 95% CI 0.15–0.85, *p* < 0.0001 and aOR 0.42, 95% CI 0.23–0.78, p < 0.0001 respectively). However, women who experienced a stillbirth were more likely to report an unusual period of baby’s movements in the prior two weeks (“once you were aware of your baby's usual pattern of movement, was there any time your baby's movements were unusual?”). The unusual pattern was typically a significant reduction in fetal activity (40% of stillbirths vs. 8.4% controls, aOR 14.1 95% 7.27–27.45, *p* < 0.0001). However, a significant increase in fetal activity compared to the usual pattern was also reported (5% of stillbirths vs. 2.4% of controls, aOR 5.60, 95% CI 1.69–18.49, *p* < 0.0001) (Table 2).

**Table 2:** Maternal report of practices of monitoring fetal movements and their perception of fetal movements during the last 2 weeks of pregnancy

Question	Response	Group				Unadjusted OR (95% CI)	Adjusted OR^*^ (95% CI)	P-value
		Cases		Controls				
		Total	%	Total	%			
During this pregnancy did your healthcare provider tell you about or ask you to keep track of your baby’s movement?	No	79	54.9	161	41.6	Reference	Reference	0.008
	Yes	65	45.1	226	58.4	0.59 (0.4, 0.86)	0.55 (0.36, 0.86)	
Did you keep track of your baby’s movement during this pregnancy?	No	70	48.3	132	33.8	Reference	Reference	0.005
	Yes	75	51.7	259	66.2	0.55 (0.37, 0.8)	0.54 (0.35, 0.83)	
How would you describe this baby’s usual movements?	Less than average movement	14	9.59	24	6.17	1.56 (0.77, 3.18)	2.21 (0.99, 4.98)	0.054
	Average movements	73	50	195	50.1	Reference	Reference	
	Above average movements	47	32.2	134	34.5	0.94 (0.61, 1.44)	0.90 (0.56, 1.44)	
	Constant movement	12	8.22	36	9.25	0.89 (0.44, 1.80)	0.98 (0.55, 2.11)	
Once you were aware of your baby’s usual pattern of movement, was there any time your baby’s movements were unusual?	No	27	19.3	200	52.5	Reference	Reference	<.0001
	Yes, a little bit less	35	25	96	25.2	2.7 (1.55, 4.72)	2.82 (1.52, 5.24)	
	Yes, significantly less	56	40	32	8.4	12.9 (7.17, 23.4)	14.13 (7.27, 27.45)	
	Yes, a little bit more	15	10.7	44	11.6	2.53 (1.24, 5.14)	2.61 (1.20, 5.66)	
	Yes, significantly more	7	5	9	2.36	5.76 (1.98, 16.7)	5.60 (1.69, 18.49)	
During the last two weeks of this pregnancy, did the STRENGTH of your baby’s movements	Stay the same	66	46.5	180	49.2	Reference	Reference	<.0001
	Decrease	58	40.9	56	15.3	2.83 (1.78, 4.49)	2.53 (1.51, 4.23)	
	Increase	18	12.7	130	35.5	0.38 (0.21, 0.67)	0.42 (0.23, 0.78)	
During the last two weeks of this pregnancy, did the FREQUENCY of your baby’s movements....	Stay the same	65	44.8	223	59.6	Reference	Reference	<.0001
	Decrease	73	50.3	76	20.3	3.29 (2.16, 5.03)	2.97 (1.86, 4.72)	
	Increase	7	4.83	75	20.1	0.32 (0.14, 0.73)	0.36 (0.15, 0.85)	
Did you usually feel your baby move at bedtime during this pregnancy?	No	5	3.42	16	4.15	Reference	Reference	0.58
	Yes	141	96.6	370	95.9	1.22 (0.44, 3.39)	1.37 (0.45, 4.23)	
Did you feel your baby move at bedtime on the last night of this pregnancy?	No	49	39.8	23	6.67	Reference	Reference	<.0001
	Yes	74	60.2	322	93.3	0.11 (0.06, 0.19)	0.11 (0.06, 0.21)	
During the last two weeks of this pregnancy, did you notice any time that your baby was more vigorous than usual)?	No	59	42.8	143	40.2	Reference	Reference	<.0001
	Yes, once.	42	30.4	24	6.74	4.24 (2.36, 7.62)	4.30 (2.25, 8.24)	
	Yes, sometimes.	30	21.7	158	44.4	0.46 (0.28, 0.75)	0.44 (0.25, 0.75)	
	Yes, often.	7	5.07	31	8.71	0.55 (0.23, 1.31)	0.71 (0.28, 1.81)	
Did you experience your baby having hiccup like movements?	No	28	20	69	18.1	Reference	Reference	0.09
	Yes	112	80	313	81.9	0.88 (0.54, 1.43)	0.61 (0.34, 1.08)	
If you noticed your baby having hiccup like movements, how long would each episode last on average?	Less than 5 min	44	40.7	160	52.5	Reference	Reference	0.17
	5 or more than 5 min	64	59.3	145	47.5	1.60 (1.02, 2.50)	1.42 (0.86, 2.36)	
If your baby experienced hiccup like movements, how often did you notice them?	Once or twice throughout this pregnancy	12	11.2	59	19.3	Reference	Reference	0.08
	Weekly	33	30.8	113	37.1	1.43 (0.69, 2.98)	1.05 (0.48, 2.31)	
	Daily	62	57.9	133	43.6	2.29 (1.15, 4.56)	1.83 (0.87, 3.88)	

^*^Adjusted by maternal age, ethnicity, smoking status, BMI, parity, country of residence, gestation, placental site

A single episode of excessive fetal movements was more frequently reported in pregnancies ending in stillbirth compared to controls (30.4% vs. 6.7%; aOR 4.30, 95% CI 2.25–8.24 p < 0.0001), there was no difference in the frequency of excessive movements between women who experienced a stillbirth in the preterm period and those whose babies died at term (26.8% of preterm cases had single episode of vigorous movement vs. 32.5% of term cases, *p* = 0.47). Whereas controls were more likely to indicate that their baby was “sometimes” vigorous compared to stillborn cases (44.4% vs. 21.7%; aOR 0.44, 95% CI 0.25–0.75 *p* < 0.0001). Likewise, increased frequency of fetal movements over the last two weeks of the pregnancy was more common in healthy controls than stillbirth (aOR 0.36, 95% CI 0.15–0.85 p < 0.0001) (Table 2).

Whilst there was no significant difference between cases and controls regarding their perception of fetal activity at bedtime during the pregnancy (96.6% v 95.9%, aOR 1.37, 95% CI 0.45–4.23 *p* = 0.58), women who had a stillbirth were significantly less likely to feel their baby move at bedtime on the last night of their pregnancy (aOR 0.11, 95% CI 0.06–0.21 *p* < 0.0001) (Table 2).

Women were asked three questions about fetal hiccups; whether they occurred, their frequency, and their duration. The presence of hiccups did not differ between cases and controls (80% vs. 81.9%). In unadjusted analyses women who experienced a stillbirth were more likely to perceive frequent (daily) hiccup occurrence (OR 2.29, 95% CI 1.15–4.56) or prolonged episodes of hiccups lasting more than five minutes (OR 1.60, 95% CI 1.02–2.50), although after adjustment for the previously mentioned variables these relationships were no longer statistically significant (aOR 1.83, 95% CI 0.87–3.88, *p* = 0.08 and aOR 1.42, 95% CI 0.86–2.36, *p* = 0.17). When birth weight percentile was included in the model for controls with complete birthweight percentile there was no difference in the results (Additional file 1: Table S1).

### Comment data analysis

The majority of respondents provided free text responses in addition to their yes/no responses to the questionnaire. For example, of the 75 parents who had a stillbirth who monitored fetal movements, 70 provided a free text response to the question “Describe how you kept track of fetal movements”. Similarly, 229 of the 259 controls who responded positively also provided a free text response. It should also be noted that each free text option had a different response rate and that the percentages in each box do not add to 100% as some participant responses fell equally into two of the identified themes and some of the responses were not common enough to fall into one of the recurring words or phrases identified.

There were five main ways participants wrote that they kept track of fetal movement (Table 3). Of those who kept track, there were no differences between the methods used to track fetal movements between cases and controls except that cases more frequently reported “active baby/constant movement” and made comments such as “*knew about kick counts but the baby was so active, I did not need to track the actual count.*”

**Table 3:** Summary of the comment analysis on free text responses

Description of how participants who responded “yes” to keeping track, kept track of fetal movements	Number of times used Cases *n* = 70 n (%)*	Number of times used Controls *n* = 229 n (%)*	Exemplar quotes
Kick Counting	39 (55.7)	149 (65.1)	• I did kick counts every day at about 8 am and 7:30 pm (Case). • Kick counts in the morning or evening. Timed how long it took to get to 10 movements (Control).
General awareness	18 (25.7)	53 (23.1)	• I just made mental note of it each day and I got use to her movements throughout the day and night (Case). • Kept a mental record of movements and discussed them with husband but did not write anything down (Control).
‘Stimulating’ the baby Drinking and eating (water/juice/sugary), music	10 (8.5)	25 (10.9)	• I did not had any method to keep track of baby movement just sit after eating and listening music or drink something like that will make him move (Case). • Just made sure I remembered when I felt her move and if I felt her throughout the day. If I didn’t/couldn’t remember movement, I’d sit still and drink some juice to see if she would wake for me (Control).
Used an i-device/app	4 (5.7)	24 (10.5)	• Baby kick app on iPhone (Case). • Kick counting app. Count ten kicks, records time between kicks and overall time to get to ten kicks (Control).
Active baby/Constant movement	5 (7.1)	3 (1.3)	• I felt her move constantly (Case). • I never had to do kick counts because I felt my baby move so much (Control).
Free text response to “Once you were aware of your baby’s usual pattern of movement, was there any time your baby’s movements were unusual?”	Number of times used Cases *n* = 75 n (%)	Number of times used Controls *n* = 118 n (%)	Exemplar quotes
Slowing down	42 (56)	13 (11)	• In the last couple of weeks of my pregnancy, I noticed the movements did slow down (Case). • In the last month before losing the baby, I felt gradual reduced movement (Case). • Slowed down at end of pregnancy (Control).
One or two quiet days	0 (0)	42 (35.6)	• One day I was not feeling well – baby moved less than normal. I went in for a non-stress test and all checked out fine (Control). • Only once and was checked by midwife (Control).
Time of day (Night time, morning, evening)	20 (26.6) (less active)	14 (11.9) (more active)	• Sometimes he wouldn’t be as active as early in the morning (Case). • The baby had specific times when she was more active (9 pm through 1 am)(Control).
Free text response to “During the last two weeks of this pregnancy, did the STRENGTH of your baby’s movements…”	Number of times used Cases n = 20 n (%)	Number of times used Controls *n* = 55 n (%)	Exemplar quotes
Less strong	8 (40)	7 (12.7)	• Slow, weak movements (Case). • Less strong in last 4 days (Case). • Less frequent and less intensity (Control).
Stronger/harder	1 (5)	22 (40)	• She was slower, but stronger (Case). • Her movements are gradually getting stronger (Control). • His kicks have gotten a little more power behind them recently (Control).
Roll/ing	4 (20)	2 (3.6)	• More of a rolling rather than punching and kicking (Case). • She seemed to roll around more during the last week (Case). • Movements are more rolls and pushes now rather than kicks and punches (Control).
Pressure/pushing/poking	0 (0)	5 (9)	• More pressure to his movements (Control). • I can feel more ‘poking’ as the baby flexes more (Control). • More pressure when poking feet/knees out (Control). • Less kicking, more pushing (Control).
Free text response to “During the last two weeks of this pregnancy, did the FREQUENCY of your baby’s movements....”	Number of times used Cases n = 23 n (%)*	Number of times used Controls n = 38 n (%)*	Exemplar quotes
Decrease	18 (78)	11 (28.9)	• Noticeable decrease in movements (Case). • Slightly fewer movements at 40 weeks (Control).
More/increased	0 (0)	10 (26.3)	• Lots of movement 1 and 2 days prior to birth (Control). • I am feeling her more due to her size (Control). • Able to feel the kicks more often, probably because they are stronger (Control).
Very Active	0 (0)	3 (7.9)	• Very active baby facing out so I’d get lots of kicks (Control).
Free text response to “During the last two weeks of this pregnancy, did you notice any time that your baby was more vigorous than usual?”	Number of times used Cases *n* = 31 n (%)	Number of times used Controls *n* = 74 n (%)	Exemplar quotes
Crazy/wild	15 (48)	1 (1.35)	• There were a few times that I mentioned to my husband how “wild and active” our baby was. I thought he was going to jump out a couple of times (Case). • The evening of the very last night he was totally crazy active. I was reassured because I thought he had been slow and sluggish for the last couple of weeks (Case). • Felt almost like she was having a seizure (Case).
Vigorous at night	*14 (45.2)* *(Noticeably vigorous night before death)*	25 (33.8) *(Normally vigorous at night)*	• Always very active, last night before she passed she was extremely active (Case). • The last night I felt him move, he was moving lots!!! More than usual. Then nothing more (Case). • His movements tend to be stronger at the end of the day when I relax on the couch around 8 pm (Control). The baby always seems to become most active when I lay down at night for bed (Control).
More powerful/strong/aggressive	0 (0)	11 (14.9)	• More powerful movements (Control). • When I was in the bath one night the movements were very strong and very low. It was quite an unusual feeling (Control). • When I sit in certain positions, she became more vigorous (Control). • A friend was feeling him move and he got really aggressive, the strongest movement I have felt him have but it was just once (Control).

*Note percentages do not add to 100%

When participants were asked “once you were aware of your baby’s usual pattern of movement, was there any time your baby’s movements were unusual?” there was a stark difference noted between responses between cases and controls (Table 3). Cases used words that indicated that their baby moved less towards the end of the pregnancy much more often than controls (56% vs. 11%). Another noticeable difference was that the cases indicated that there were certain times of the day that their baby was *less* active. Conversely, the controls indicated that their baby was *more* active at certain times of day. The controls were also much more likely to report in the qualitative analysis that their baby had one or two quiet days than the cases (34.7% vs. 8%, Table 3).

When participants were asked to provide comment on change in strength in fetal movements (“during the last two weeks of this pregnancy, were the strength of your baby’s movements…..”) 40% of the cases reported that their baby’s movements were less strong, using words like “slow” and “weak”, compared to only 12.7% of controls. In contrast, 40% of the controls reported that their baby’s movements were stronger using words like “powerful” and “stronger”, compared to only 5% of cases. In addition, only the controls used words like, “pressure”, “poking”, and “pushing” or said that they could actually see their baby moving. On the other hand, cases used words like “rolling” to describe a change in strength.

When asked to describe a change in frequency in fetal movements (“during the last two weeks of this pregnancy, did the frequency of your baby’s movements…..”), many more cases than controls used words that indicated a decrease in frequency (78% vs. 28.9%). For example, the cases said things like “*notable decrease in movements*” unlike controls who described “*slightly fewer movement*s”. The controls typically described an increase in the frequency of movements using words like *“I am feeling her more due to her size”.* Notably, none of the descriptions the (*n* = 23) cases gave, reported an increase unless they were reporting a change from increase to decrease “*Increased until 5 days prior when she started to move less”.* (Table 3).

When asked to describe a time when their baby had more vigorous movement (“during the last two weeks of this pregnancy, did you notice any time that your baby was more vigorous than usual?”) 48% of the cases who gave further detail described a single occasion of increased vigour and used words like “frantic” or “crazy” to describe this time, whereas only one of the controls used this kind of word. Interestingly, this control gave details of this experience:

“the night he was born there were violent kicks. I went to the hospital because it was quite painful. I was told that I needed an emergency c-section. The cord was wrapped around his neck. He was quite purple but ended up being just fine.”

Conversely, controls used words like “powerful”, “strong” and “aggressive” to describe times of increased vigour and tended not to provide a description about a single incident of increased vigour, rather they made comments suggesting that increased vigour was a typical pattern in the previous two weeks, for example; “The baby always seems to become most active when I lay down at night for bed’ (Table 3).

## Discussion

### Main findings

This study provides further evidence that stillbirth is associated with a change in fetal movements. However, while reduced fetal movements are known to be a risk for stillbirth [16, 17], we have provided novel data that a sudden change in vigour – described as “crazy”, “wild” or “frantic” movements - were almost exclusively reported by women who had a stillbirth. This exaggerated activity can be differentiated from a general increase in fetal movements in the preceding weeks which was a reassuring feature. Importantly, while women who had a stillbirth were less likely to have been tracking fetal movements, they were also significantly less likely to report having been instructed by a care provider to keep track. These findings are in agreement with other research studies and a recent Confidential Enquiry into Antepartum Stillbirths [5, 6, 18]. Together these data highlight the importance of care providers having a documented discussion to encourage women to become familiar with their baby’s pattern of movement and to respond to changes in fetal activity.

### Strengths and limitations

A strength of this international nested-case control study is the series of questions about fetal movements that has allowed exploration of patterns in fine detail in order to gain a more comprehensive understanding of fetal movement in the weeks prior to a stillbirth (or previous weeks of pregnancy for control women). The inclusion of free-text sections following each question enabled a descriptive analysis of women’s experiences in their own words that allowed the emergence of recurring words rather than yes/no responses to questions assumed to be important from the investigator’s perspective. We focused on late stillbirths (28 weeks gestation or later) in order to examine novel and potentially modifiable risk factors that could identify fetuses otherwise able to survive *ex utero*. This approach also minimised the inclusion of responses from a mother whose stillbirth could be associated with a serious anomaly. The use of an internet-based questionnaire meant that participants did not come from a single site which increases the generalisability of the findings.

Despite its strengths our approach had several limitations. Firstly, use of an online questionnaire prevented selection of matched controls for each stillbirth. Nonetheless, while matching may improve study efficiency it can also have important disadvantages since it may be impossible to find an exact match especially in a study that has a time-sensitive element (e.g., late pregnancy); this can subsequently render the study inefficient. Additional inefficiencies can emerge when the matching variable is not a true confounder. Recall bias could be considered as a limitation which may have affected mothers’ memory of whether they were informed about fetal movements or their memory of fetal movement. Critically, the study methodology attempted to reduce recall bias in several important ways. Firstly, all women were asked the same series of questions that also included open-ended responses. The case group comprised only women who had recently (within the previous 30 days) experienced a stillbirth, a time when events surrounding the death can be clearly recalled [19]. Moreover, 73% of the control women were still pregnant at the time of survey completion and a further 23% completed the survey within 3 weeks of delivery making it unlikely that experiences or concerns would be systematically biased in terms of outcomes affecting reported experiences. Also, since this study revealed novel findings, recall bias is unlikely to have been influenced by information available to mothers. A final limitation is the possibility of misclassification. While we could confirm a live birth for the majority of control women, two control women subsequently delivered a stillborn baby after completion of the survey during pregnancy. This is not unexpected given the current stillbirth rate in high-income countries [20]. Although there may be other babies that died which were not reported to us, the expected number would be very small, and would not influence the results seen here. Furthermore, the sensitivity analysis demonstrated that excluding women with unknown outcomes did not significantly alter the study findings.

There may be other sources of bias which should be considered. Women who had experienced a stillbirth in a previous pregnancy may have been motivated to participate in a subsequent healthy pregnancy; these women may experience pregnancy differently. It is possible that women who had poor pregnancy outcomes did not answer the follow up questionnaire accurately leading to ascertainment bias. However, this seems not to have been the case as in addition to including at least 2 perinatal losses, the group of control women reported a variety of birth outcomes (data not shown) which indicate that not all control pregnancies were uncomplicated.

### Interpretation

While the relationship between reduced fetal movements and adverse pregnancy outcome has been described since the 1970s [21], the impact of a single episode of excessive fetal movements is a relatively novel finding that has been rarely reported or investigated. In a historical, uncontrolled, cohort of women who experienced a stillbirth we have previously documented that 16.7% of women reported increased movement, however it was not clear in this study if this was a single episode or not [6].Moreover, our findings have provided further support for those of Stacey et al. who also reported that a single episode of vigorous activity was associated with an almost 7-fold increase in late stillbirth (aOR: 6.81; 95% CI: 3.01–15.41). Similarly, our findings of increase in strength, frequency or multiple episodes of vigorous fetal movements was protective against stillbirth were also reported by Stacey et al. Importantly, the protective effect of a gradual increase in strength and frequency do not contradict the finding that an isolated event of vigorous movement was found to be associated with stillbirth. Indeed, it is probable that the increase in strength and frequency are reflective of a general trend in fetal behaviour across the previous two weeks but an isolated event that is uncharacteristic for the fetus may be a warning sign.

Understanding the relationship between altered fetal movements and stillbirth is complicated by an appreciation of the time of fetal death. For example, a single period of increased fetal activity may be associated with a terminal event only diagnosed in retrospect. The qualitative comments participants provided in this study identified a common scenario almost exclusively described by women who experienced a stillbirth. This was a period when their fetus went “crazy” or “wild”. It was noted by Sadovsky in the 1970’s that any “…sudden, strong, vigorous fetal movements with increased rate followed by cessation was almost invariably a sign of acute fetal distress and fetal death and may indicate fetal distress and an hypoxic-ischaemic insult” [21]. Sadovsky goes on to speculate that this movement may be the fetus’ attempt to “release a complication” such as cord entanglement, if the movements were “successful in releasing the complication then normal fetal movements would resume, if they did not, fetal demise usually resulted.” Women in our study often reported this kind of movement in the days and hours prior to their baby’s death suggesting that if the woman appreciates this as a warning sign of a baby in trouble that there may be a short window of opportunity for her to seek immediate care and possible intervention to prevent fetal death. This seems particularly pertinent given that the only control who described this episode sought immediate care and required an emergency Caesarean section. These unique descriptors in the comment data also provide maternity care providers with the words women may use to describe acute fetal compromise and differentiate this from normal periods of increased fetal activity associated with reduced odds of stillbirth in both this study and Stacey el al.’s study.

In this study women with stillbirths experienced a gradual slowing of fetal movements towards the end of pregnancy, with reported reductions in the strength and frequency of movements and a number of case babies had a single period of excessive movement describe as “crazy” or “wild” in the hours or days before their death. Conversely, controls described more activity and stronger movements as pregnancy progressed that became more powerful to the point that they could be seen externally. Control responses indicated that it may be normal for the well fetus to have one or two quiet days during the pregnancy and that some babies had regular times for increased vigorous activity. Therefore, listening and responding to these keywords could provide maternity care providers with a window of opportunity to identify and manage the fetus at increased risk of stillbirth.

Studies have highlighted that women frequently receive inconsistent information regarding what to do in the case of reduced fetal movements [22] and the information obtained via the internet is highly variable and frequently inaccurate [23]. This study highlights that women who experienced a stillbirth reported that they were less likely to be counselled regarding the importance of fetal movements and were less likely to monitor fetal movements, reprising concerns about the lack of education given to mothers about the significance of reduced fetal movements described in Confidential Enquiries into Antepartum Stillbirths [18, 24]. The recent Stillbirth Priority Setting Partnership identified relevant research priorities in this field, particularly whether more accessible evidence-based information on signs and symptoms of stillbirth risk (in this case altered fetal movements) would reduce the incidence of stillbirth [25]. Further studies are needed to determine accurate information and the most effective timing and means of delivery to inform women about what to do after perceiving alterations in fetal movements. In addition, in this and the cohort study some women who had received education about fetal movements did not report reduced fetal movements to their maternity care provider. Thus, further study is also needed regarding the barriers and facilitators for women seeking advice from their care provider for abnormal fetal movements.

## Conclusions

In summary, this study confirms that all pregnant women need to be educated that a reduction in fetal activity should be reported to their maternity care provider. Additionally, if the woman notices a sudden period of increased vigour that she would describe as “crazy” or “wild,” this should also be considered a significant event which should be reported. Conversely, this study suggests that a single day of reduced movements may be normal for the well fetus and that a gradual increase in strength and multiple episodes of vigorous movements described as “powerful” and “aggressive” also appear to be reassuring for fetal health.

We report a protective effect in participants who “kept track” of fetal movements. However, there was little difference in the descriptions that participants used as to how they kept track, aside from the fact there was a slightly higher percentage of cases than controls who indicated that their baby moved so much they felt they did not need to keep track. This may suggest that whatever method the mother uses that it is important for her to get to know her baby’s individual pattern of movement, because this allows her an opportunity to report significant changes in her baby’s behaviour to her care provider. However, more research is warranted into assessing which means of tracking fetal movements provides the most accessible information for women to enable her to get to know her unborn baby and thus allow her to immediately report changes in her unborn baby’s behaviour to maximise the care providers’ ability to keep her and her unborn baby safe. In addition, better management, which is evidence-based, is needed to improve management and detection of the fetus at risk due to changes in fetal movement.

## Additional file

Additional file 1: Table S1. Maternal report of practices of monitoring fetal movements and their perception of fetal movements during the last 2 weeks of pregnancy comparing controls with known birth weight and the total population (ZIP 19 kb)

## Abbreviations

aOR: adjusted Odds Ratio
CI: Confidence Interval
OR: Odds Ratio
STARS: Study of Trends and Associated Risks for Stillbirth
