# Supplementary material for: Stillbirth is associated with perceived alterations in fetal activity – findings from an international case control study
Source: BMC Pregnancy Childbirth. 2017 Nov 13;17:369. doi: 10.1186/s12884-017-1555-6 (PMC5683455; doi:10.1186/s12884-017-1555-6)
Supplement: Additional file 1: Table S1. — Maternal report of practices of monitoring fetal movements and their perception of fetal movements during the last 2 weeks of pregnancy comparing controls with known birth weight and the total population (ZIP 19 kb) [file 12884_2017_1555_MOESM1_ESM.zip › ESM/12884_2017_1555_MOESM1_ESM.docx]

**Table S1** Maternal report of practices of monitoring fetal movements and their perception of fetal movements during the last 2 weeks of pregnancy comparing controls with known birth weight and the total population.

| **Question** | **Response** | **Unadjusted OR (95% CI)** | **Adjusted OR***  **(95% CI)** | **P-value** | **Number of Observations** | **Subgroup analysis on those with birthweight centile** | | |
| --- | --- | --- | --- | --- | --- | --- | --- | --- |
|  |  |  |  |  |  | **OR** (95% CI)** | **P-value**** | **Number of Observations** |
| During this pregnancy did your healthcare provider tell you about or ask you to keep track of your baby's movement? | No | Reference | Reference | 0.008 | 502 | Reference | 0.147 | 331 |
|  | Yes | 0.59  (0.4, 0.86) | 0.55  (0.36, 0.86) |  |  | 0.64  (0.36, 1.17) |  |  |
| Did you keep track of your baby's movement during this pregnancy? | No | Reference | Reference | 0.005 | 505 | Reference | 0.016 | 332 |
|  | Yes | 0.55  (0.37, 0.8) | 0.54  (0.35, 0.83) |  |  | 0.47  (0.26, 0.87) |  |  |
| How would you describe this baby's usual movements? | Less than average movement | 1.56  (0.77, 3.18) | 2.21  (0.99, 4.98) | 0.054 | 505 | 3.24  (0.95, 11.6) | 0.204 | 332 |
|  | Average movements | Reference | Reference |  |  | Reference |  |  |
|  | Above average movements | 0.94  (0.61, 1.44) | 0.90  (0.56, 1.44) |  |  | 0.82  (0.42, 1.58) |  |  |
|  | Constant movement | 0.89  (0.44, 1.80) | 0.98  (0.55, 2.11) |  |  | 1.34  (0.47, 3.80) |  |  |
| Once you were aware of your baby's usual pattern of movement, was there any time your baby's movements were unusual? | No | Reference | Reference | <.0001 | 491 | Reference | <.0001 | 323 |
|  | Yes, a little bit less | 2.7  (1.55, 4.72) | 2.82  (1.52, 5.24) |  |  | 1.93  (0.85, 4.43) |  |  |
|  | Yes, significantly less | 12.9  (7.17, 23.4) | 14.13  (7.27, 27.45) |  |  | 15.17  (5.88, 39.13) |  |  |
|  | Yes, a little bit more | 2.53  (1.24, 5.14) | 2.61  (1.20, 5.66) |  |  | 1.72  (0.57, 5.15) |  |  |
|  | Yes, significantly more | 5.76  (1.98, 16.7) | 5.60  (1.69, 18.49) |  |  | 8.00  (1.41, 45.52) |  |  |
| During the last two weeks of this pregnancy, did the STRENGTH of your baby's movements | Stay the same | Reference | Reference | <.0001 | 481 | Reference | 0.005 | 324 |
|  | Decrease | 2.83  (1.78, 4.49) | 2.53  (1.51, 4.23) |  |  | 3.01  (1.40. 6.50) |  |  |
|  | Increase | 0.38  (0.21, 0.67) | 0.42  (0.23, 0.78) |  |  | 0.73  (0.33, 1.59) |  |  |
| During the last two weeks of this pregnancy, did the FREQUENCY of your baby's movements.... | Stay the same | Reference | Reference | <.0001 | 493 | Reference | 0.006 | 328 |
|  | Decrease | 3.29  (2.16, 5.03) | 2.97  (1.86, 4.72) |  |  | 2.50  (1.27, 4.92) |  |  |
|  | Increase | 0.32  (0.14, 0.73) | 0.36  (0.15, 0.85) |  |  | 0.50  (0.18, 1.43) |  |  |
| Did you usually feel your baby move at bedtime during this pregnancy? | No | Reference | Reference | 0.58 | 503 | Reference | 0.68 | 330 |
|  | Yes | 1.22  (0.44, 3.39) | 1.37  (0.45, 4.23) |  |  | 1.50  (0.22, 10.11) |  |  |
| Did you feel your baby move at bedtime on the last night of this pregnancy? | No | Reference | Reference | <.0001 | 441 | Reference | <0.0001 | 292 |
|  | Yes | 0.11  (0.06, 0.19) | 0.11  (0.06, 0.21) |  |  | 0.10  (0.04, 0.26) |  |  |
| During the last two weeks of this pregnancy, did you notice any time that your baby was more vigorous than usual)? | No | Reference | Reference | <.0001 | 469 | Reference | <0.0001 | 316 |
|  | Yes, once. | 4.24  (2.36, 7.62) | 4.30  (2.25, 8.24) |  |  | 4.20  (1.61, 10.98) |  |  |
|  | Yes, sometimes. | 0.46  (0.28, 0.75) | 0.44  (0.25, 0.75) |  |  | 0.45  (0.22, 0.94) |  |  |
|  | Yes, often. | 0.55  (0.23, 1.31) | 0.71  (0.28, 1.81) |  |  | 1.01  (0.29, 3.55) |  |  |
| Did you experience your baby having hiccup like movements? | No | Reference | Reference | 0.087 | 495 | Reference | 0.61 | 325 |
|  | Yes | 0.88  (0.54, 1.43) | 0.61  (0.34, 1.08) |  |  | 0.81  (0.37, 1.80) |  |  |
| If you noticed your baby having hiccup like movements, how long would each episode last on average? | Less than 5 minutes | Reference | Reference | 0.17 | 391 | Reference | 0.29 | 251 |
|  | 5 or more than 5 min | 1.60  (1.02, 2.50) | 1.42  (0.86, 2.36) |  |  | 1.43  (0.74, 2.77) |  |  |
| If your baby experienced hiccup like movements, how often did you notice them? | Once or twice throughout this pregnancy | Reference | Reference | 0.08 | 391 | Reference | 0.35 | 250 |
|  | Weekly | 1.43  (0.69, 2.98) | 1.05  (0.48, 2.31) |  |  | 1.57  (0.55, 1.48) |  |  |
|  | Daily | 2.29  (1.15, 4.56) | 1.83  (0.87, 3.88) |  |  | 2.05  (0.78, 5.55) |  |  |

*Adjusted by maternal age, ethnicity, smoking status, BMI, parity, country of residence, gestation, placental site

**Adjusted by maternal age, ethnicity, smoking status, BMI, parity, country of residence, gestation, placental site, birthweight percentile
